# Effect of Commercial Cyanobacteria Products on the Growth and Antagonistic Ability of Some Bioagents under Laboratory Conditions

**DOI:** 10.1155/2013/838329

**Published:** 2013-11-07

**Authors:** Nehal S. El-Mougy, Mokhtar M. Abdel-Kader

**Affiliations:** Plant Pathology Department, National Research Centre, El-Behoose Street, Dokki, Giza 12662, Egypt

## Abstract

Evaluation of the efficacy of blue-green algal compounds against the growth of either pathogenic or antagonistic microorganisms as well as their effect on the antagonistic ability of bioagents was studied under *in vitro* conditions. The present study was undertaken to explore the inhibitory effect of commercial algal compounds, Weed-Max and Oligo-Mix, against some soil-borne pathogens. In growth medium supplemented with these algal compounds, the linear growth of pathogenic fungi decreased by increasing tested concentrations of the two algal compounds. Complete reduction in pathogenic fungal growth was observed at 2% of both Weed-Max and Oligo-Mix. Gradual significant reduction in the pathogenic fungal growth was caused by the two bioagents and by increasing the concentrations of algal compounds Weed-Max and Oligo-Mix. The present work showed that commercial algal compounds, Weed-Max and Oligo-Mix, have potential for the suppression of soil-borne fungi and enhance the antagonistic ability of fungal, bacterial, and yeast bio-agents.

## 1. Introduction

Several commercially available products have shown significant disease reduction through various mechanisms to reduce pathogen development and disease. Different approaches may be used to prevent, mitigate, or control plant diseases. Beyond good agronomic and horticultural practices, growers often rely heavily on chemical fertilizers and pesticides. Such inputs to agriculture have contributed significantly to the spectacular improvements in crop productivity and quality over the past 100 years. However, the environmental pollution caused by excessive use and misuse of agrochemicals, as well as fear-mongering by some opponents of pesticides, has led to considerable changes in people's attitudes towards the use of pesticides in agriculture. Application of biological control using antagonistic microorganisms has proved to be successful for controlling various plant diseases [1]. However, it is still not easy and costly in application. It can serve as the best control measure under green-house conditions. The concern of pesticide use with respect to human health and environment has brought increasing interest in alternatives by avoiding negative effects on the environment. Today, there are strict regulations on chemical pesticide use, and there is political pressure to remove the most hazardous chemicals from the market. Recently algal are one of the chief biological agents that have been studied for the control of plant pathogenic fungi, particularly soil-borne disease [2]. For example, cyanobacteria (blue-green algal) and eukaryotic algal produce biologically active compounds that have antifungal activity [3, 4] and antibiotic and toxic activity [5, 6] against plant pathogens. *Anabaena* spp. [7, 8], *Scytonema* spp. [9], and *Nostoc* spp. [10] were shown to be efficient in the control of damping-off as well as the growth of the soil fungus *Cunninghamella blakesleeana*. In particular, culture filtrates or cell extracts from cyanobacteria and algal applied to seeds protect them from damping-off fungi such as *Fusarium* sp., *Pythium* sp., and *Rhizoctonia solani *[3]. The present research focuses on finding compounds that are safe to humans and the environment, for example, algal as well as biocontrol agents which may provide an alternative control of many soil and seed-borne pathogens. The objective of the present work was to evaluate the effect of algal compounds against the growth of pathogenic fungi as well as bioagents and their antagonistic ability as well.

## 2. Materials and Methods

Evaluation an integrated treatments using bioagents and algal compounds against cucumber, tomato and pepper root rot pathogens as well as disease incidence was carried out under laboratory, greenhouse, and plastic house conditions.

### 2.1. Tested Materials

*(a) Root Rot Pathogens. *Root rot fungal pathogens are *Alternaria solani*, *Fusarium solani*, *F. oxysporum*, *Rhizoctonia solani*, *Sclerosium rolfsii*, *Sclerotinia sclerotiorum,* and *S. minor*. These seven pathogens were isolated from various root rotted vegetables grown under commercial plastic houses located at different areas at Egypt [11].

*(b) Antagonistic Microorganisms.* The bioagents fungal antagonists, that is, *Trichoderma harzianum*, *T. viride*, and *T. hamatum*, as well as bacterial antagonists, that is, Bacillus subtilis, Pseudomonas fluorescens, and the yeast Saccharomyces cerevisiae, obtained from the Culture Collection Unit of,  Plant Pathology Deptartment, National Research Centre, Egypt were used in present work. These bioagents proved their high antagonistic effect against a wide spectrum of plant pathogens in many previous works at the same department.

*(c) Commercial Algal Compounds*. Purchased commercial algal compounds, that is, Weed-Max (cyanobacteria extracts in powder phase) produced by Inc. Trade S.A.E. Company, Naser City, Egypt, and Oligo-X algal (blue-green algal extracts in liquid phase) produced by the Arabian Group for Agricultural Service, 114 King Fesal Street, Giza, Egypt, were used in the present study. 

### 2.2. Laboratory Tests

The inhibitory effect of Weed-Max and Oligo-X (cyanobacteria extracts) against the growth of the root pathogenic fungi, fungal, bacterial, and yeast antagonists as well as antagonistic ability of the bioagents (fungi-bacteria) was evaluated *in vitro* using the culture technique [12]. *In vitro *studies of tested microorganisms were performed on PDA medium in 9 cm diameter Petri dishes. 

The tested soil-borne pathogenic fungi were *Alternaria solani*, *Fusarium solani*, *F. oxysporum*, *Rhizoctonia solani*, *Sclerotium rolfsii*, *Sclerotinia sclerotiorum*, and* S. minor*. Meanwhile, the tested antagonistic microorganisms (fungi-bacteria-yeast) were *Trichoderma harzianum*, *T*. *viride*, *T. hamatum*, *Bacillus subtilis*, *Pseudomonas fluorescens*, and* Saccharomyces cerevisiae*. 

#### 2.2.1. Effect on Fungal and Bacterial Growth

Tested algal extracts were added to conical flasks containing sterilized PDA medium before its solidifying to obtain the proposed concentrations of 0.5, 1.0, and 2.0% and rotated gently to ensure equal distribution of added algal extracts. A separate PDA flask free of tested algal extracts was used as control treatment. The supplemented media were poured into sterilized Petri dishes (9 cm Ø) approximately 20 mL each. Mycelial discs (5 mm Ø) taken from the periphery of an actively growing PDA culture of each tested fungus were placed at the centre of the prepared Petri dishes then incubated for seven days at 25 ± 2°C. Five replicates were used for each treatment. The average linear growth diameter of colonies was measured, and reduction in fungal growth was calculated relative to the controls. All tests were repeated three times. The bacterial bioagents, that is, *Bacillus subtilis*, *Pseudomonas fluorescens*, and* Saccharomyces cerevisiae*, were grown on nutrient broth medium, while yeast was grown on NYDB medium [13]. All tested bacteria and yeast were incubated in a rotary shaker at 200 rpm for 24 h at  28   ±  2°C. The bacterial and yeast cells were harvested by centrifugation at 6,000 rpm for 10 min, washed twice with 0.05 M phosphate buffer at pH 7.0, and resuspended in distilled water. The concentrations of bacterial and yeast cells in the suspensions were adjusted to 1 × 10^4^ cells per milliliter (colony forming unit/mL) with the aid of a haemocytometer slide in order to determine cells counts. The inhibitory effect of tested algal extracts on colony formed by antagonistic bacterial and yeast isolates was assayed in nutrient broth media using a modified method described earlier [14]. Aliquots of 100 *μ*L of the bacterial cells suspension (1 × 10^4^) were transferred to glass tubes (180 × 16 mm) containing 10 mL sterilized distilled water; then the tested materials were added individually to each tube to achieve the proposed concentration. All tubes were left for 12 h then shaked well using Vortex for 5 min. One mL of each test tube was dispensed into Petri dishes, and about 20 mL of semisolidifying sterilized agar medium (nutrient or NYPD) was poured into the inoculated dishes and rotated gently to ensure equal distribution of the bacterial inocula. Controls were the bacteria or yeast cells suspension free from tested algal extracts. All inoculated and controls dishes were incubated for 72 h at 28 ± 2°C and then examined. Percent of bacterial and yeast isolates formed colonies was calculated by comparing them with their counts in controls. All treatments consisted of five replicates, and experiments were repeated three times.

#### 2.2.2. Effect on the Antagonistic Ability of Biocontrol Agents

The effect of algal extracts on the antagonistic ability of the fungal, bacterial, and yeast antagonistic agents against the linear growth of the root pathogenic fungi was evaluated under *in vitro *conditions.

Antagonistic studies of biocontrol microorganisms against pathogenic fungi were performed on PDA medium in 9 cm diameter Petri dishes. Abundant fungal, bacterial, and yeast growth was prepared. For bacterial and yeast inoculum, ten mL of each individual bacterial and yeast isolate was grown for 48 h on nutrient or NYPD broth media and poured into flasks containing sterilized PDA medium. Before solidifying, each flask was rotated gently to ensure equal distribution of bacterial or yeast growth and then poured into 9 cm diameter Petri dishes. Inoculated plates were incubated for 48 h at 28 ± 2°C. A 5 mm disk of each bacterial or yeast growth was used for antagonism test. For fungal growth, 5 mm disk of each tested fungus was transferred to the centre of a PDA dish and then incubated at 28 ± 1°C. The incubation period was 5 and 7 days for antagonistic and pathogenic fungi, respectively. *In vitro*, antagonistic studies of biocontrol microorganisms (all lower case) and pathogenic fungi were performed on PDA medium in 9 cm diameter Petri dishes supplemented with different concentrations of the tested materials. A 5 mm disk of each antagonistic fungal, bacterial, or yeast growth culture was placed onto the PDA, 10 mm from the edge of the Petri dish. Another disk of the same diameter of each pathogenic fungal growth culture was placed on one side of the dish at the same distance and used for general control. The control treatment was inoculated with a culture disk of either pathogenic or antagonistic culture alone at the same conditions in medium free of algal compounds. Both experimental and control dishes were assigned to a completely randomized design, with five replicates per treatment. The antagonistic effect measurements were carried out when the pathogenic fungal growth filled all Petri dishes in general control. All inoculated Petri dishes were incubated at 28 ± 2°C, and the fungal growth diameter away from and towards the antagonist agent was measured after the pathogenic fungal growth in the control treatment had reached the edge of the Petri dish [15]. This test was repeated three times, and the inhibition was calculated as the percentage reduction in colony diameter of pathogenic fungal growth compared with its growth in control for each particular tested bioagent. All *in vitro *adjustment of supplemented media with different concentration of tested chemicals as well as fungal inoculation, incubation conditions, and growth measurements and calculations were followed as stated before.

### 2.3. Statistical Analysis

All experiments were set up in a complete randomized design. ANOVA was used to analyze differences between antagonistic inhibitor effect and linear growth of pathogenic fungi *in vitro*. A general linear model option of the analysis system SAS [16] was used to perform the ANOVA. The MSTAT-C program (V2.1) was used to perform the analysis of variance. The Tukey test for multiple comparisons among means was utilized [17].

## 3. Results and Discussion

Many strategies to control the fungal pathogens have been investigated in the field [18, 19]. Currently, the most effective method in preventing soil-borne diseases is to apply chemical fungicides which could be harmful to other living organisms and reduce useful soil microorganisms [20, 21]. Therefore, public concern is focused on alternative methods of pest control, which can play a role in integrated pest management systems to reduce dependence on chemical pesticides [22]. 

### 3.1. Effect on Fungal and Bacterial Growth

The effect of algal compounds on the growth of either pathogenic or antagonistic bioagents is presented in Tables 1 and 2. Presented data in Table 1 reveal that all tested fungi were affected positively with the two algal compounds. Also, it was observed that the inhibitory effect on fungal growth increased as the concentrations of algal compounds are also increased. The highest reduction in pathogenic fungal growth, at treatment of 2% Weed-Max, was recorded as a range between 50.0 and 64.4%, meanwhile, a range of 13.3–20.0% was recorded for fungal antagonists. On the other hand, treatment of 2% Oligo-X caused the highest reduction in pathogenic fungal growth and ranged between 54.4–63.3% and 12.2–17.7% for antagonistic fungi. As for determination of different concentrations of either Weed-Max or Oligo-X on the viability of antagonistic bacteria and yeast, the obtained results are presented in Table 2. Presented data showed similar effect of algal compounds on the viability of both bacteria and yeast. Data revealed that bacteria and yeast viability started significantly to be affected with both algal compounds at their concentrations of 1 or 2%. The highest reduction in bacterial viability was recorded as 15.5, 17.5 and 17.4, 20.3% for *B. subtilis* and *P. fluorescens*, at 3% of Weed-Max and Oligo-X treatments, respectively. Meanwhile, 10.5 and 13.3% was recorded as the highest reduction in *S. cerevisiae* viability at the same concentration of both algal compounds, in respective order.

### 3.2. Effect on the Antagonistic Ability of Biocontrol Agents

**Figure 1:**
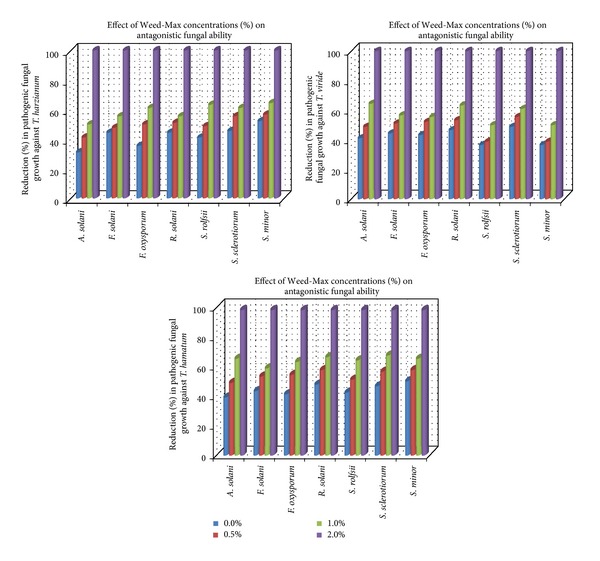
Reduction (%) in pathogenic fungal growth against *Trichoderma* spp. in medium supplemented with Weed-Max at different concentrations *in vitro*.

**Figure 2:**
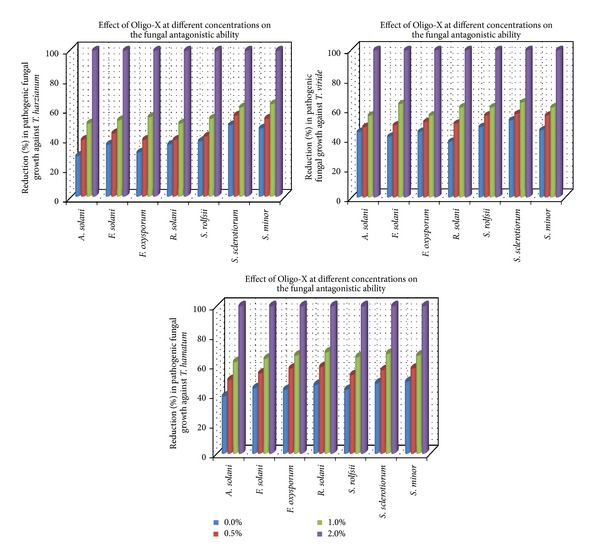
Reduction (%) in pathogenic fungal growth against *Trichoderma* spp. in medium supplemented with Oligo-X at different concentrations *in vitro*.

Evaluation of the efficacy of tested commercial algal products on the antagonistic ability of some bioagents *in vitro* is presented in Table 3 and Figures 1 and 2. Data in Table 3 show that in the presence of the antagonists (*Trichoderma* spp.) the linear growth of pathogenic fungi reduced gradually by increasing concentrations of either Weed-Max or Oligo-Mix. Complete inhibition of pathogenic fungal growth was recorded at the highest concentration using 2% of both algal compounds. Also, the antagonistic ability of *Trichoderma* spp. was enhanced by the addition of algal compounds to the growth medium. In general, it was observed that the measured linear growth of pathogenic fungi fluctuated against the tested *Trichoderma* spp. *T. hamatum *seems to be*  *more effective against the pathogenic fungal growth followed by *T. viride* and *T. harzianum*, respectively.

Moreover, the average linear growth of pathogenic fungi against *T. harzianum* ranged between 43 and 62 mm and against *T. viride* was 46–53 mm while against *T. hamatum* was 44–54 mm. This range reduced to be 32–45, 32–45, and 28–36 mm at the concentration of 1% of Weed-Max as well as 33–45, 32–40, and 28–34 mm at concentration 1% of Oligo-Mix, in respective order. 

On the other hand, the illustrated data in Figures 1 and 2 show a parallel reduction in the growth of pathogenic fungi against the antagonistic fungi *Trichoderma* spp. in correlation with the increase of used concentrations of both algal compounds. Gradual significant reduction in the pathogenic fungal growth was caused by the two bioagents and increasing the concentrations of algal compounds Weed-Max and Oligo-Mix. Complete reduction in pathogenic fungal growth was observed at 2% concentration of both algal compounds. The present work showed that commercial algal compounds, Weed-Max and Oligo-Mix, have a potential for the suppression of soil-borne fungi and enhance the antagonistic ability of fungal, bacterial, and yeast bioagents.

**Figure 3:**
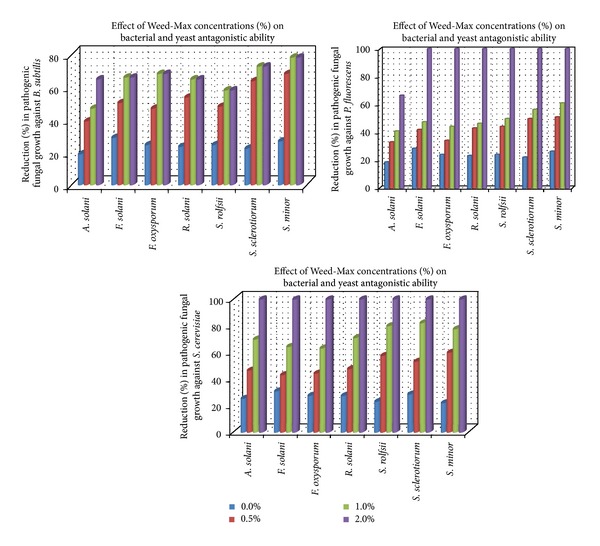
Reduction (%) in pathogenic fungal growth against bacteria and yeast in medium supplemented with Weed-Max at different concentrations *in vitro*.

**Figure 4:**
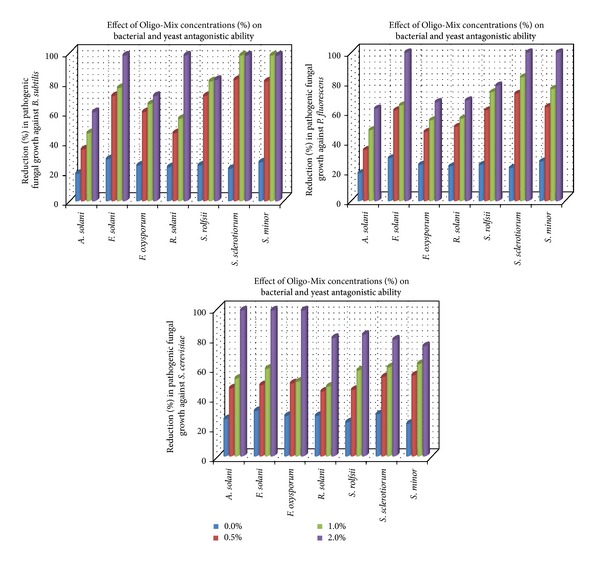
Reduction (%) in pathogenic fungal growth against bacteria and yeast in medium supplemented with Oligo-Mix at different concentrations *in vitro*.

The presented data in Table 4 reveal the enhancing effect of tested commercial algal compounds on the antagonistic ability of bioagents, bacteria and yeast *in vitro*. The antagonistic effect of *B. subtilis*, *P. fluorescence*, and* S. cerevisiae *against the growth of pathogenic fungi increased gradually by increasing added concentrations of either Weed-Max or Oligo-Mix in the growth medium. The data also show that the average linear growth of pathogenic fungi against *B. subtilis* ranged between 64 and 73 mm and against *P. fluorescence* was 64–73 mm while against *S. cerevisiae *was 16–70 mm. This range was reduced to be 20–48, 35–53, and 16–33 mm at concentration 1% of Weed-Max as well as 0–48, 15–47, and 33–47 mm at a concentration of 1% of Oligo-Mix, in respective order. These averages revealed that Oligo-Mix has more enhancing effect on the antagonistic ability of tested bacterial and yeast bioagents comparing with Weed-Max. Moreover, the yeast *S. cerevisiae* had superior antagonistic effect against pathogenic fungal growth followed by *B. subtilis *and* P. fluorescence*, respectively. On the other hand, the illustrated data in Figures 3 and 4 showed that the reduction (%) in pathogenic fungal growth was correlated to the gradual increase in concentrations of algal compounds in the growth medium. Data also show that complete inhibition of fungal growth fluctuated between high concentrations of either Weed-Max or Oligo-Mix. Concerning fungal growth reduction, complete inhibition (100%) was recorded at 2% of Weed-Max for all tested pathogenic fungi when tested against the antagonists *B. subtilis*, *P. fluorescence*, and* S. serevisiae*.

In this regard, only the fungus *A. solani *showed*  *an exception of this observation that its growth was reduced by the same concentration (2%).A similar observation was also recorded concerning the algal compound Oligo-Mix. Complete inhibition in pathogenic fungal growth was recorded at concentration of 2% for the pathogenic fungi *F. solani *and *R. solani *when grown*  *against* B. subtilis, *meanwhile the superior effect of Oligo-Mix at 1% concentration caused 100% growth reduction of *S. sclerotiorum* and *S. minor* against the same antagonist *B. subtilis.* Furthermore, at a concentration of 2% of Oligo-Mix complete inhibition (100%) in the pathogenic fungal growth was recorded only for growth of *F. solani*, *S. sclerotiorum,* and *S. minor* when grown against *P. fluorescens*, meanwhile at the same concentration 100% of growth inhibition of *A. solani*, *F. solani*, and *F. oxysporum* was observed when grown against *S. serevisiae*.

Similar results were previously recorded [23]. Laboratory experiments were carried out *in vitro*; the first was to evaluate the effect of culture filtrates of nine algal strains (*Anabaena flosaquae*, *Anabaena oryzae*, *Chlorella vulgaris*, *Nostoc muscorum*, *Nostoc humifusum*, *Oscillatoria *sp., *Phormidium fragile*, *Spirulina platensis*, and *Wollea saccata*) at concentrations of 10, 20, 30, and 40% on mycelium growth and spore production of *Cercospora beticola *causing leaf spot disease in sugar beet [23]. Generally, they found that all the algal culture filtrates reduced the fungal mycelium growth, but the best results were obtained by *Spirulina platensis*, *Oscillatoria *sp. and *Nostoc muscorum*; the highest fungal mycelium growth inhibition percentage was achieved by the concentrations of 30% (100, 100, and 82%, resp.) and at 40% (100, 100, and 100%, resp.). Fungal spore production (number of spores) was completely inhibited by the previous three algal cultural filtrates particularly at the concentration of 40%. They conclude that the antifungal activity of the algal culture filtrates has been attributed to the presence of bioactive compounds, that is, total phenolic compounds, total saponins, and alkaloids in the algal culture filtrates. Also, several workers reported that the extracts of *Nostoc muscorum* significantly inhibited the growth of *Candida albicans* and *Sclerotinia sclerotiorum *[24–27]. Moreover, *Nostoc muscorum* was effective on the growth of some plant pathogens [28]. Also it was reported that cyanobacteria filtrates strongly inhibited the phytopathogenic fungi isolated from leaves, stems, and roots of Faba bean [29]. Moreover, mycelia growth of several phytopathogenic fungi such as *Fusarium oxysporum*, *Penicillium expansum*, *Phytophthora cinnamomi*, *Rhizoctonia solani*, *Sclerotinia sclerotiorum*, and *Verticillium alboatrum* was inhibited by the methanol extracts of the *cyanobacterium Nostoc* strain ATCC 53789 [18]. It was reported that the growth of *R. solani* on PDA was significantly inhibited by using *Nostoc muscorum* extract [3]. Also, *Nostoc muscorum* filtrates were found to have potential for the suppression of phytopathogenic fungi such as the sugar beet pathogens *Fusarium verticillioides*, *Rhizoctonia solani*, and *Sclerotium rolfsii *[30]. The *in vitro* and *in vivo *growth, sporulation, and sclerotial production were significantly inhibited with *Nostoc muscorum *[30]. Furthermore, many works concerning the antimicrobial bioactive algal compounds were reported. In this regards, marine algal or seaweeds are rich and varied source of bioactive natural products so it has been studied as potential biocidal and pharmaceutical agents [31]. There have been a number of reports of antibacterial activity from marine plants [32, 33], and special attention has been reported for antibacterial and/or antifungal activities related to marine algal against several pathogens [34]. Also, seaweeds are considered as a source of bioactive compounds as they are able to produce a great variety of secondary metabolites characterized by a broad spectrum of biological activities [35] with antiviral, antibacterial, and antifungal activities [36] which act as potential bioactive compounds of interest for pharmaceutical applications [37]. Most of these bioactive substances isolated from marine algal are chemically classified as brominated, aromatics, nitrogen-heterocyclic, nitro sulphuric-heterocyclic, sterols, dibutanoids, proteins, peptides and sulphated polysaccharides [34]. The antibacterial activity of seaweeds is generally assayed using extracts in various organic solvents, for example, acetone, methanol-toluene, ether, and chloroform-methanol [38]. Using of organic solvents always provides a higher efficiency in extracting compounds for antimicrobial activity [39]. Several extractable compounds, such as cyclic poly-sulfides and halogenated compounds, are toxic to microorganisms and, therefore, responsible for the antibiotic activity of some seaweeds [40]. Moreover, the extraction of antimicrobials from the different species of seaweeds was solvent dependen. Methanol was a good solvent for extraction of antimicrobials from brown seaweeds, whereas acetone was better for red and green species [35].

In conclusion, the present *in vitro* experiments revealed the sensitivity of pathogenic fungi to algal compounds as well as the enhancing effect on the antagonistic ability of fungal, bacterial, and yeast bioagents. Algae and consequently their extracts can be the treasure trove of biologically active compounds. Their beneficial properties for humans, animals, and plants were recognized in the past and are appreciated nowadays in the development of new biotechnological products. Moreover, application of the algal extracts as the components of pharmaceuticals, feeds for animals, and fertilizers was recorded [41].

The present results lead to the suggestion that algal compounds and bioagent could be considered as promising seed or soil treatment against plant pathogenic fungi causing root diseases. These applicable treatments characterized as clean, cheep, and fungicide alternative and as being not hazardous to environment could be used against such soil-borne plant diseases. 

## Figures and Tables

**Table 1:** Effect of algal compounds on the growth of some antagonistic and pathogenic soil-borne fungi *in vitro*.

Tested fungi	Control		Weed-Max						Oligo-X					
	0.0		0.5%		1.0%		2.0%		0.5%		1.0%		2.0%	
	L* (mm)	R** %	*L* (mm)	*R* %	*L* (mm)	*R* %	*L* (mm)	*R* %	*L* (mm)	*R* %	*L* (mm)	*R* %	*L* (mm)	*R* %
*A. solani *	90^a^	0	62^c^	31.1	53^d^	41.1	45^e^	50.0	65^c^	27.7	55^d^	38.8	45^e^	50.0
*F. solani *	90^a^	0	50^d^	44.4	47^e^	47.7	40^e^	55.5	58^d^	35.5	51^d^	43.3	43^e^	52.2
*F. oxysporum *	90^a^	0	58^d^	35.5	45^e^	50.0	35^f^	61.1	63^c^	30.0	55^d^	38.8	41^e^	54.4
*R. solani *	90^a^	0	50^d^	44.4	44^e^	51.1	40^e^	55.5	57^d^	36.6	55^d^	38.8	45^e^	50.0
*S. rolfsii *	90^a^	0	53^d^	41.1	46^e^	48.8	33^f^	63.3	56^d^	37.7	53^d^	41.1	42^e^	53.3
*S. sclerotiorum *	90^a^	0	49^e^	45.5	40^e^	55.5	35^f^	61.1	46^e^	48.8	40^e^	55.5	35^f^	61.1
*S. minor *	90^a^	0	43^e^	52.2	39^f^	56.6	32^f^	64.4	48^e^	46.6	42^e^	53.3	33^f^	63.3
*T. harzianum *	90^a^	0	90^a^	0	86^a^	4.4	78^b^	13.3	90^a^	0	85^a^	5.5	79^b^	12.2
*T. viride *	90^a^	0	90^a^	0	83^a^	7.7	74^b^	17.7	90^a^	0	82^a^	8.8	78^b^	13.3
*T. hamatum *	90^a^	0	90^a^	0	80^a^	11.1	72^b^	20.0	90^a^	0	81^a^	10.0	74^b^	17.7

Mean values within columns followed by the same letter are not significantly different (*P* ≤ 0.05).

*L: linear fungal growth (mm).

**R: reduction in fungal growth calculated relatively to fungal growth in control (free of algal compounds).

**Table 2:** Effect of algal compounds on the viability of some antagonistic bacteria and yeast *in vitro*.

Algal compounds	Concentrations (%)	Antagonistic bacteria and yeast					
		*B. subtilis *		*P. fluorescens *		*S. cerevisiae *	
		CFU	*R* (%)	CFU	*R* (%)	CFU	*R* (%)
Control	0.0	148^∗a^	0.0**	157^∗a^	0.0	142^∗a^	0.0
Weed-Max	0.5	148^a^	0.0	157^a^	0.0	142^a^	0.0
	1.0	137^b^	7.4	139^b^	11.4	135^b^	4.9
	2.0	125^c^	15.5	128^c^	17.4	127^c^	10.5
Oligo-X	0.5	148^a^	0.0	157^a^	0.0	142^a^	0.0
	1.0	134^b^	9.4	136^b^	13.3	137^b^	3.5
	2.0	122^c^	17.5	125^c^	20.3	123^c^	13.3

Mean values within columns followed by the same letter are not significantly different (*P* ≤ 0.05).

*CFU: no. of colony formed unit (1 × 10^4^/mL).

**R: reduction in fungal growth calculated relatively to bacterial and yeast growth in control (free of algal extracts).

**Table 3:** Effect of Weed-Max and Oligo-X concentrations on the antagonistic ability of fungi against some soil-borne pathogenic fungi *in vitro*.

Pathogenic fungi	Antagonistic fungi											
	*T. harzianum *				*T. viride *				*T. hamatum *			
	Weed-Max concentration (%)											
	0.0	0.5	1.0	2.0	0.0	0.5	1.0	2.0	0.0	0.5	1.0	2.0
*A. solani *	62^∗f^	53^e^	45^d^	0^a^	53^e^	46^d^	32^c^	0^a^	54^e^	45^d^	30^bc^	0^a^
*F. solani *	50^e^	47^d^	40^d^	0^a^	50^e^	44^d^	39^c^	0^a^	50^e^	41^d^	36^c^	0^a^
*F. oxysporum *	58^e^	45^d^	35^c^	0^a^	51^e^	43^d^	40^d^	0^a^	52^e^	40^d^	32^c^	0^a^
*R. solani *	50^e^	44^d^	40^d^	0^a^	48^d^	42^d^	33^c^	0^a^	46^d^	37^c^	29^b^	0^a^
*S. rolfsii *	53^e^	46^d^	33^c^	0^a^	57^e^	55^e^	45^d^	0^a^	51^e^	43^d^	31^c^	0^a^
*S. sclerotiorum *	49^d^	40^d^	35^c^	0^a^	46^d^	40^d^	35^c^	0^a^	47^d^	38^c^	28^b^	0^a^
*S. minor *	43^d^	39^c^	32^c^	0^a^	57^e^	55^e^	45^d^	0^a^	44^d^	37^c^	30^bc^	0^a^

	Bio-Mix concentration (%)											
*A. solani *	62^∗f^	55^e^	45^d^	0^a^	50^e^	47^d^	40^d^	0^a^	55^e^	45^d^	34^c^	0^a^
*F. solani *	50^e^	51^e^	43^d^	0^a^	53^e^	46^d^	33^c^	0^a^	50^e^	41^d^	32^c^	0^a^
*F. oxysporum *	58^e^	55^e^	41^d^	0^a^	50^e^	44^d^	40^d^	0^a^	51^e^	38^c^	30^c^	0^a^
*R. solani *	50^e^	55^e^	45^d^	0^a^	56^e^	45^d^	35^c^	0^a^	48^d^	37^c^	28^b^	0^a^
*S. rolfsii *	53^e^	53^e^	42^d^	0^a^	47^d^	40^d^	35^c^	0^a^	51^e^	42^d^	31^c^	0^a^
*S. sclerotiorum *	49^d^	40^d^	35^c^	0^a^	43^d^	39^c^	32^c^	0^a^	47^d^	39^c^	29^b^	0^a^
*S. minor *	43^d^	42^d^	33^c^	0^a^	49^d^	40^d^	35^c^	0^a^	46^d^	38^c^	30^c^	0^a^

Mean values within columns followed by the same letter are not significantly different (*P* ≤ 0.05).

*Linear fungal growth (mm).

**Table 4:** Effect of Weed-Max and Oligo-X on the antagonistic ability of bacteria and yeast against some soil-borne pathogenic fungi *in vitro*.

Pathogenic fungi	Antagonistic bacteria and yeast											
	*B. subtilis *				*P. fluorescence *				*S. cerevisiae *			
	Weed-Max concentrations (%)											
	0.0	0.5	1.0	2.0	0.0	0.5	1.0	2.0	0.0	0.5	1.0	2.0
*A. solani *	73^a^	55^c^	48^d^	32^e^	73^a^	60^b^	53^c^	30^e^	67^b^	48^d^	27^f^	0^h^
*F. solani *	64^b^	45^d^	31^e^	0^h^	64^b^	52^c^	47^d^	0^h^	62^b^	51^c^	32^e^	0^h^
*F. oxysporum *	68^b^	48^d^	29^f^	0^h^	68^b^	59^c^	50^c^	0^h^	65^b^	50^c^	33^e^	0^h^
*R. solani *	69^b^	42^d^	32^e^	0^h^	69^b^	51^c^	48^d^	0^h^	69^b^	47^d^	26^f^	0^h^
*S. rolfsii *	68^b^	47^d^	38^e^	0^h^	68^b^	50^c^	45^d^	0^h^	64^b^	38^e^	18^g^	0^h^
*S. sclerotiorum *	70^a^	33^e^	25^f^	0^h^	70^a^	45^d^	39^e^	0^h^	63^b^	42^d^	16^g^	0^h^
*S. minor *	66^b^	29^f^	20^f^	0^h^	66^b^	44^d^	35^e^	0^h^	70^b^	36^e^	20^f^	0^h^

	Oligo-X concentrations (%)											
*A. solani *	73^∗a^	58^c^	48^d^	35^e^	73^a^	59^c^	47^d^	34^e^	67^b^	48^d^	42^d^	0^h^
*F. solani *	64^b^	25^f^	20^f^	0^h^	64^b^	35^e^	32^e^	0^h^	62^b^	46^d^	36^e^	0^h^
*F. oxysporum *	68^b^	35^e^	30^e^	25^f^	68^b^	48^d^	41^d^	30^e^	65^b^	45^d^	44^d^	0^h^
*R. solani *	69^b^	48^d^	39^e^	0^h^	69^b^	45^d^	40^d^	29^f^	69^b^	50^d^	47^d^	17^g^
*S. rolfsii *	68^b^	25^f^	16^g^	15^g^	68^b^	35^e^	24^f^	20^f^	64^b^	49^d^	37^e^	15^g^
*S. sclerotiorum *	70^a^	15^g^	0^h^	0^h^	70^a^	25^f^	15^g^	0^h^	63^b^	41^d^	35^e^	18^g^
*S. minor *	66^b^	61^b^	0^h^	0^h^	66^b^	33^e^	22^f^	0^h^	70^b^	40^d^	33^e^	22^f^

Mean values within columns followed by the same letter are not significantly different (*P* ≤ 0.05).

*Linear fungal growth (mm).
